# Recipient Tregs: Can They Be Exploited for Successful Hematopoietic Stem Cell Transplant Outcomes?

**DOI:** 10.3389/fimmu.2022.932527

**Published:** 2022-06-21

**Authors:** Sabrina N. Copsel, Dietlinde Wolf, Brent Pfeiffer, Henry Barreras, Victor L. Perez, Robert B. Levy

**Affiliations:** ^1^ Department of Microbiology and Immunology, University of Miami School of Medicine, Miami, FL, United States; ^2^ Sylvester Comprehensive Cancer Center, University of Miami School of Medicine, Miami, FL, United States; ^3^ Department of Pediatrics, University of Miami School of Medicine, Miami, FL, United States; ^4^ Foster Center for Ocular Immunology, Duke Eye Center, Duke University, Durham, NC, United States; ^5^ Department of Ophthalmology, University of Miami School of Medicine, Miami, FL, United States

**Keywords:** Tregs, GvHD, HSCT = hematopoietic stem cell transplant, recipient tregs, treatment

## Abstract

Human and mouse CD4^+^FoxP3^+^ T cells (Tregs) comprise non-redundant regulatory compartments which maintain self-tolerance and have been found to be of potential therapeutic usefulness in autoimmune disorders and transplants including allogeneic hematopoietic stem cell transplantation (allo-HSCT). There is substantial literature interrogating the application of donor derived Tregs for the prevention of graft versus host disease (GVHD). This Mini-Review will focus on the recipient’s Tregs which persist post-transplant. Although treatment in patients with low dose IL-2 months post-HSCT are encouraging, manipulating Tregs in recipients early post-transplant is challenging, in part likely an indirect consequence of damage to the microenvironment required to support Treg expansion of which little is understood. This review will discuss the potential for manipulating recipient Tregs *in vivo prior to* and *after* HSCT (fusion proteins, mAbs). Strategies that would circumvent donor/recipient peripheral blood harvest, cell culture and *ex-vivo* Treg expansion will be considered for the translational application of Tregs to improve HSCT outcomes.

## Introduction

CD4^+^FoxP3^+^ Tregs have demonstrated immune regulatory activity which can provide therapeutic application to allogeneic transplantation including hematopoietic stem cell transplants (HSCT). Their use in experimental as well as clinical transplants has shown the ability to promote engraftment and diminish both host versus graft (HVG) and graft versus host (GVH) responses (1–5). To date, the majority of experimental studies have focused on donor - *not recipient* - Tregs as strategies to reduce allo-immune responses and promote tolerance post-HSCT (1, 6–12). Some earlier studies did adoptively transfer “recipient” Tregs obtained from syngeneic animals and demonstrated their capacity to ameliorate acute and chronic GVHD (13–15).

Interestingly, manipulation of Tregs in recipients of clinical HSCT with chronic GVHD has shown encouraging results; however, the precise origin of these Tregs within the patients (i.e. donor versus recipient) was not a focus of the study (3, 12). Following transplant, the source of Tregs is variable and may include transplanted mature and subsequently *de novo* derived populations of both donor and recipient origin (**Table 1**) derived from persisting hematopoietic precursor cells (16, 17). Immediately post-transplant, recipient Tregs have been identified by a number of investigators and their manipulation has been reported to ameliorate GVHD (2, 18). In the present review, we will focus on recipient Tregs and their ability and potential use to regulate GVHD following allo-HSCT. Specifically, recipient Tregs (rTregs) will be defined as those cells persisting/surviving following conditioning and transplant. We posit rTregs have been underappreciated to date. Strategies to optimize and exploit their regulatory capacity in different recipient tissues may lead to novel translational strategies for improving allo-HSCT outcomes.

**Table 1:** Sources of Tregs in recipients before and after hematopoietic stem cell transplants.

Potential sources of Tregs present in recipients of HSCT	Populations of Tregs present in recipients at the time-period *in vivo* treatment administered:	
	Pre-conditioning and transplant	Post-transplant
***Donor origin (dTregs)***		
Transplanted mature Tregs	Not applicable	Yes
*De novo* derived Tregs from transplanted stem cells	Not applicable	Yes
***Recipient origin (rTregs)***		
Mature Tregs	Yes	Yes (“persisting”)
*De novo* derived from surviving stem cells	Yes	Yes

## Recipient Treg Presence and Function Following Allogeneic HSCT

**Figure 1:**
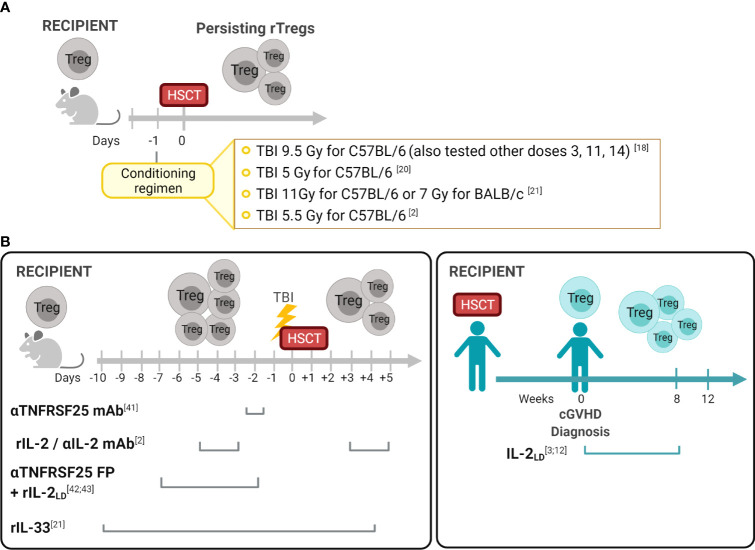
Recipient Treg persistence and manipulation in allogeneic hematopoietic stem cell transplantation **(A)** Persisting Tregs (rTregs) have been identified after different conditioning protocols in recipients post-autologous or T cell depleted allo-HSCT (18). These findings established the opportunity to consider manipulating this population before and/or after transplants to diminish GVHD and improve overall outcomes. **(B)** Manipulation of the Treg compartment in experimental and clinical HSCT. In contrast to the post-transplant period when both rTreg and dTreg populations could be present in recipients (**Table 1**), in the pre-transplant period only rTregs can be regulated. Reagents which engage receptors on Tregs were utilized *in vivo* to activate these cells and diminish GVHD. As noted, some treatments were administered only in the pre-transplant period (anti-TNFRSF25mAb +/- rIL-2; IL-2/αIL-2mAb) and another pre and early post-HSCT (IL-33). Notably, studies in clinical HSCT recipients administered the reagent (rIL-2) only following transplant and GVHD diagnosis. Created with *BioRender.com*.

During the last 15 years, several groups including our own have reported identifying recipient Tregs (rTregs) post-HSCT which possess functional suppressive capacity *in vivo*. Early studies by Shlomchik and colleagues found that radiation-resistant recipient T cells ameliorated GVHD in a chronic murine model (14). Experiments demonstrated that this process was mediated only by persisting host CD4^+^CD25^+^ but not CD4^+^CD25^-^ host T cells (14). Studies by our laboratory definitively demonstrated persisting or “residual” rTregs following varying conditioning levels using syngeneic transplants with congenic markers to discriminate donor and recipient populations (18). The results following allogeneic T cell depleted grafts were similar to the syngeneic HSCT results, i.e. rTregs persisted and comprised a higher frequency of surviving CD4 T cells after sublethal and lethal TBI conditioning. Notably, some rTregs persisted weeks after transplant, underwent a significant expansion dependent primarily on IL-2, and possessed suppressive function inhibiting T cell proliferation *in vitro* and providing *in vivo* protection against development of autoimmune disease (18, 19). Other reports also noted Tregs exhibit radiation resistance resulting in increased FoxP3^+^ frequency within the animal’s CD4 compartment (20, 21). Qu et al. found that Tregs were more resistant to gamma TBI (5 Gy) compared to CD4 Tconv cells. This observation was accompanied by a higher Bcl-2 expression in Tregs. Although persisting Tregs from irradiated mice exhibited suppressive ability, their function was slightly reduced when compared to Tregs from non-irradiated mice (20). In studies utilizing scurfy bone marrow chimeras, restoration of the peripheral Treg pool was found to be contributed to by radioresistant host cells (22). Studies examining resistance to bone marrow engraftment following MHC-matched allo-HSCT found rTregs after conditioning (5.5 Gy) and transplant which could respond to subsequent activating (IL-2) signals (discussed below) (2) (**Figure 1A**). In total, these pioneering studies raised the notion that such recipient cells could be useful in regulating transplant outcomes.

Importantly, several clinical studies have noted the persistence of tissue resident recipient T cells in patients following HSCT. For example, a recent clinical investigation reported the presence of a significant frequency of host T cells up to a year post-HSCT in transplant recipient tissues, like colon and skin but not peripheral blood (23, 24). Based on T cell clonal analyses pre- and post-HSCT, the authors interpreted these cells as persisting/surviving recipient populations. Interestingly, similar to the experimental findings concerning Tregs post-HSCT (2, 18, 19), these human recipient CD4 T cells were present in patients who received myeloablative or nonmyeloablative conditioning (myeloablative, median 22%, range 6%–87%; nonmyeloablative, median 12%, range 7%–41%) (23). The authors proposed that CD25 and Ki67 expression on host T cells as well as expression of IFN-γ and IL-17 *in situ* evidenced their activated and functional state and hypothesized that these host T cells contributed to tissue GVHD (23). Similarly, an independent investigation characterized radio/chemotherapy-resistant tissue-resident memory T cells in the skin of patients following allo-HSCT including FoxP3^+^ Tregs which contained a small subset of CD69^+^ FoxP3^+^ cells suggesting an activated status (24). Analysis of oral mucosal tissue in patients found that FoxP3^+^ cells were observed in the basement membrane in acute (small numbers of FoxP3^+^CD8^+^ T cells) and chronic GVHD patients, although the origin of these Tregs were not examined (25). A recent study examining blood following autologous HSCT for non-Hodgkin lymphoma patients reported that high levels of proliferating Tregs within a month post-transplant correlated with higher relapse rates. The investigators speculated that the rapid expansion of these regulatory cells resulted from either the host’s residual T cells or *via* donor cells from the graft (26).

## Manipulation of Tregs in Recipients of HSCT

Administration of reagents directed at activating Tregs post-HSCT are thought to be primarily targeting transplanted, i.e. donor Tregs (dTregs). As described above, studies from a number of laboratories have shown that rTregs persist following a variety of conditioning levels including some ablative strategies and transplant and hence could be manipulated as well (2, 18, 20). While transplanted dTregs have been spared direct effects of the conditioning regimens, both persisting rTregs together with dTregs ‘find themselves’ present within a microenvironment which has undergone varying levels of damage dependent on the type and aggressiveness of the conditioning regimen employed. A limited clinical study reported only a transient Treg increase following IL-2 administration early post-HSCT, however Tregs levels fell and no improvement in GVHD was observed (27). Notably, it was possible to expand and activate rTregs using an rIL-2/αIL-2 complex following a reduced intensity conditioning (5.5 gy TBI) protocol (2). Therefore, we posit that in addition to providing appropriate signaling *via* surface receptors on Tregs which are present immediately post-transplant, other signals from the microenvironment are likely required for effective expansion and functional activation. To circumvent these issues, Treg stimulation prior to the conditioning and transplant may provide a novel approach to reduce GVHD and improve outcomes.

Many studies have reported strategies administering reagents into mice to manipulate Tregs *in vivo*, reviewed in (28). Targeting cell surface receptors such as CD25 (IL-2Rα), TNFRSF1B (TNFR2), TNFRSF4 (OX-40), ST-2 (IL-33R) and TNFRSF25 (DR3) by injection of mAbs and fusion proteins can expand Tregs in both hematolymphoid and other compartments, including GVHD target tissues i.e. GI tract, skin, and eye (6, 7, 21, 29–39). Driving Treg expansion *via* these molecules enabled investigations interrogating how their application could be applied to recipients undergoing HSCT. Preclinical findings led to clinical studies demonstrating *in vivo* administration of low-dose recombinant human (rIL-2_LD_) into patients’ months post-HSCT with diagnosed cGVHD, increased their peripheral blood Treg levels and function which correlated with reduced cutaneous GVHD (3, 40, 41). These studies were not directed to determine the source of the Tregs responding to rIL-2_LD_ which could have included each of the populations described in **Table 1**. Importantly, early experimental studies from our laboratory examined resistance to bone marrow engraftment following MHC-matched allo-HSCT under sub-lethal conditioning regimens and reported that administration of rIL-2/αIL-2 mAb complex before transplantation induced activation and expansion of rTregs resulting in a transient increase of donor hematopoietic engraftment (2) (**Figure 1B**). More recently, IL-33 administration peri-allo-HSCT increased levels of ST2 expressing immune cells including rTreg. This regulatory population was demonstrated to persist after high intensity conditioning mediating the protection against acute GVHD following MHC-mismatched allo-HSCT (21). Furthermore, IL-33-expanded rTregs regulated macrophage activation and suppressed effector T cell infiltration of the small intestine – a key GVHD-target tissue (21). TNFR2 activation before allo-HSCT was also shown to expand radiation resistant rTregs resulting in improved overall survival and decreased of GVHD severity (32). Another member of the TNFR superfamily, TNFRSF25, was demonstrated to effectively expand rTegs in the context of BMT. Nishikii and colleagues demonstrated that prophylactic administration of a single injection of αTNFRSF25 mAb to recipient mice pre-HSCT increased rTregs, but not dTregs, leading to a significant reduction of GVHD (42). However, using the same mAb post-transplant promoted GVHD by inducing activation and proliferation of donor T cells (42). Lastly, our laboratory recently demonstrated that targeting TNFRSF25, with an agonistic fusion protein (TL1A-Ig), and rIL-2_LD_ prior to conditioning alone or with transplant (8.5 Gy, **Figure 1B**), significantly increased rTreg levels (both FoxP3^+^Rorγt^-^ and FoxP3^+^Rorγt^+^) in the lamina propria of the large intestine 4 days post-allo-HSCT which persisted up to 10 days (43). Importantly, this preclinical prophylactic regimen with TL1A-Ig+Il-2_LD_ enhanced GVHD overall survival in an MHC-mismatched allo-HSCT model (43, 44).

In total, findings to date demonstrate that Tregs can be manipulated in recipients before and following transplant. The type of transplant (allogeneic vs autologous) and level of conditioning (myeloablative vs reduced intensity) will impact the effectiveness of pre- and post-transplant Treg stimulation. For example, following aggressive conditioning and allo-HSCT, Treg manipulation may be delayed due to an impaired microenvironment needing time to reconstitute. In contrast, targeting of the rTreg compartment prior to transplant can circumvent these events enabling potent expansion of these cells. Furthermore, the presence of increased levels of activated effector T cells post-transplant likely would decrease the selective targeting of Tregs by the reagents discussed. Lastly, in contrast to applying the beneficial effects of only peripheral blood dTregs, treatment of recipients is not restricted to circulating populations but includes all tissues comprising key targets of GVHD.

## FUTURE APPLICATION OF HOST TREGS TO CLINICAL HSCT

As mentioned above, recent clinical studies have reported the presence and survival of host T cells in GVHD target tissues post-conditioning and transplant and posited such cells may contribute to GVHD (23, 24). These findings together with experimental work identifying rTregs early post-HSCT indicate that some lymphoid cells do survive chemo/radio insults and can also undergo subsequent division when the appropriate signals are present. While the mechanisms which underlie the persistence and survival of Tregs post-HSCT have not been formally identified, CD137 (41BB), a survival signal is expressed on Tregs (45). Increased levels of anti-apoptotic signals have been reportedly increased in Tregs after TBI (20) and ligand signaling during inflammation can elevate bcl-2, bcl-x2 and survivin (46, 47). After administration of cyclophosphamide post-transplant, elevated ALDH in Tregs as noted may afford some protection from alkylation effects (48, 49). Although we are not presently aware of reports in the literature, it is possible that following conditioning protocols using cyclophosphamide (typically combined with TBI), persisting rTregs may be present. Interestingly, EGFR has been shown to be expressed on Tregs under inflammatory conditions and contributes to their optimal function (50). Amphiregulin/EGFR signaling was reported to protect hematopoietic stem cells post-irradiation exposure *via* augmenting DNP-kinase activity, promoting DNA repair pathways (51). The abundant presence of amphiregulin in the GI tract and skin, together with the inflammatory milieu post-conditioning, might therefore promote rTregs survival through a similar amphiregulin/EGFR signaling pathway (52–54).

Based on the overall findings reported to date, we speculate that exploiting Treg suppression systemically together with such cells within GVHD target tissues will provide the highest likelihood to successfully ameliorate acute GVHD during the early post-transplant period. Evidence supports the notion that Tregs in hematolymphoid compartments versus tissues, including the skin and GI, differ in part due to their microenvironment. For example, Tregs at barrier sites are phenotypically distinct from their lymphoid-organ counterparts, and such ‘tissue’ signatures can reflect their tissue-adapted function. This could result from metabolic processes dependent on local substrate availability in part regulated by microbiota (55). Recipient Tregs physiologically present in the transplant recipient’s tissues may be thought of as “in place” and therefore could be effective immediately at the outset of transplantation. To test such a hypothesis, strategies need to be designed to manipulate and take advantage of both systemic and tissue resident populations. Regarding the former, donor Tregs included in the transplant inoculum enter the recipient systemic circulation and have been shown to rapidly access hematolymphoid compartments post-infusion (56). Such infused dTregs are not directly exposed to conditioning and therefore can provide, a) an efficient ‘therapy’ to regulate the peripheral hematolymphoid compartments immediately post-transplant together with b) the potential for self-renewing Treg populations contained within the CD62L^+^KLRG1^-^ central “cTreg” compartment (57, 58).

Some rTregs clearly persist post-HSCT, including within GVHD target tissues (2, 13, 18, 43), however little is known regarding how they may be manipulated to provide optimal tissue regulation, decrease inflammation, and lymphoid suppression early post-transplant. Negrin and colleagues administered an anti-TNFRSF25 mAb two days prior to HSCT and reported diminished GVHD (42). The investigators proposed this result occurred as a consequence of increased peripheral Tregs, which presumably involved rTregs although Tregs in recipient tissues were not examined in the study. Based on antibody half-life, it’s direct stimulation of donor Tregs might also have contributed to the improved outcomes (**Figure 1B**). Pre- and post-HSCT IL-33 infusion (D-10➔+4) reportedly increased Treg levels in the colon 2-3 weeks post-transplant with a significant component expressing ST2 (21). Notably, this strategy diminished GVHD and prolonged recipient survival (**Figure 1B**). Our group reported that administering an agonistic fusion protein (FP) containing the TNFRSF25 ligand, TL1A, for 4 days (Day -7 to Day -4) followed by low dose IL-2 (Days -4, -3, -2) prior to conditioning and HSCT, resulted in dramatic improvement in animal survival following MHC-mismatched experimental HSCT (43, 44). The short half-life (hours) of the αTNFRSF25 FP (13 hrs.) compared to the mAb (5 days) virtually eliminated the likelihood of its presence post-HSCT so donor T cell populations would not be stimulated (59). Marked increases in rTreg frequency and numbers were identified in the colon of these animals up to 2 weeks post-transplant (43, 44).

All Tregs present in recipients post-transplant are subject to the extant microenvironment which nurtures and maintains homeostasis of the compartment. Our laboratory and others have struggled to successfully manipulate Tregs *in situ* early post-HSCT and we posit this is a consequence of conditioning induced damage to the Treg micro-environment and the requisite signals required to promote their activation, differentiation, and proliferation. We believe studies are urgently needed to identify and define the key elements of this environment and importantly, the kinetics of its reconstitution following allogeneic as well as syngeneic HSCT. The varying conditioning regimens (radiation, chemo, Abs. etc.) are likely to differentially disrupt the microenvironment as well as influence the kinetics of its repair and rebound. Based on our own work, we postulate that 3-4 weeks is minimally needed to “rejuvenate/re-build” the Treg microenvironment under moderately aggressive conditioning regimens. Although identifying the key signals can ultimately lead to efforts to minimize/protect the microenvironment from conditioning induced damage, developing strategies to target Tregs prior to conditioning currently provides an excellent opportunity to exploit the rTregs before environmental disruption and early findings suggest such manipulations can improve overall survival and function early post-HSCT.

## Author Contributions

SC and RL outlined and wrote the review article. DW, BP, HB, and VP read, edited and critiqued the manuscript. All authors contributed to the article and approved the submitted version.

## Funding

This work was supported by funds from the National Institutes of Health (R01 EY024484-06, R01 EY030283-01: RBL, VLP and R41 AI149916-01: RBL), and the Sylvester Comprehensive Cancer Center (RBL). SC is the recipient of a ASTCT New Investigator Award. Research reported in this publication was also supported by the National Cancer Institute (Award P30CA240139).

## Author Disclaimer

The content is solely the responsibility of the authors and does not necessarily represent the official views of the National Institutes of Health.

## Conflict of Interest

RL is a compensated consultant/advisory board member for and equity holder in Heat Biologics and consultant for Kimera Labs. Neither are directly relevant to this review.

The remaining authors declare that the research was conducted in the absence of any commercial or financial relationships that could be construed as a potential conflict of interest.

## Publisher’s Note

All claims expressed in this article are solely those of the authors and do not necessarily represent those of their affiliated organizations, or those of the publisher, the editors and the reviewers. Any product that may be evaluated in this article, or claim that may be made by its manufacturer, is not guaranteed or endorsed by the publisher.
